# Healthy Lifestyle and Risk of Cancer in the European Prospective Investigation Into Cancer and Nutrition Cohort Study

**DOI:** 10.1097/MD.0000000000002850

**Published:** 2016-04-22

**Authors:** Fiona McKenzie, Carine Biessy, Pietro Ferrari, Heinz Freisling, Sabina Rinaldi, Veronique Chajès, Christina C. Dahm, Kim Overvad, Laure Dossus, Pagona Lagiou, Dimitrios Trichopoulos, Antonia Trichopoulou, H. Bas Bueno-de-Mesquita, Anne May, Petra H. Peeters, Elisabete Weiderpass, Maria-Jose Sanchez, Carmen Navarro, Eva Ardanaz, Ulrika Ericson, Elisabet Wirfält, Ruth C. Travis, Isabelle Romieu

**Affiliations:** From the International Agency for Research on Cancer (IARC), Lyon (FM, CB, PF, HF, SR, VC, IR), INSERM, Centre for Research in Epidemiology and Population Health (CESP) (LD), Paris South University, UMRS 1018 (LD), Department of Public Health, Aarhus University, Aarhus, Denmark (CCD, KO), Institute Gustave Roussy (LD), INSERM, Centre for Research in Epidemiology and Population Health (CESP), Villejuif, France (LD), Department of Hygiene, Epidemiology and Medical Statistics, University of Athens Medical School (PL), Bureau of Epidemiologic Research, Academy of Athens (PL, DT), Hellenic Health Foundation, Athens, Greece (DT, AT), Department of Epidemiology, Harvard School of Public Health, Boston, MA (PL, DT), National Institute for Public Health and the Environment (RIVM), Bilthoven (HBBM), Department of Gastroenterology and Hepatology, University Medical Centre (HBBM), Julius Center, University Medical Center Utrecht, Utrecht, The Netherlands (AM, PHP), School of Public Health, Imperial College, London (HBBM, PHP), Cancer Epidemiology Unit, University of Oxford, Oxford, United Kingdom (RCT), Faculty of Medicine, University of Malaya, Kuala Lumpur, Malaysia (HBBM), Department of Community Medicine, Faculty of Health Sciences, UiT, The Arctic University of Norway, Tromsø (EW), Department of Research, Cancer Registry of Norway, Oslo, Norway (EW), Department of Medical Epidemiology and Biostatistics, Karolinska Institutet, Stockholm (EW), Department of Clinical Sciences in Malmö, Lund University, Lund, Sweden (UE, EW), Samfundet Folkhälsan, Helsinki, Finland (EW), CIBER Epidemiology and Public Health (CIBERESP), España (M-JS, CN, EA), Andalusian School of Public Health, University of Granada, Granada (M-JS), Department of Epidemiology, Murcia Regional Health Council (CN), Department of Health and Social Sciences, Universidad de Murcia, Murcia (CN), Navarra Public Health Institute (EA), and Navarra Institute for Health Research (IdiSNA), Pamplona, Spain (EA).

## Abstract

It has been estimated that at least a third of the most common cancers are related to lifestyle and as such are preventable. Key modifiable lifestyle factors have been individually associated with cancer risk; however, less is known about the combined effects of these factors.

This study generated a healthy lifestyle index score (HLIS) to investigate the joint effect of modifiable factors on the risk of overall cancers, alcohol-related cancers, tobacco-related cancers, obesity-related cancers, and reproductive-related cancers. The study included 391,608 men and women from the multinational European Prospective Investigation into Cancer and Nutrition (EPIC) cohort. The HLIS was constructed from 5 factors assessed at baseline (diet, physical activity, smoking, alcohol consumption, and anthropometry) by assigning scores of 0 to 4 to categories of each factor, for which higher values indicate healthier behaviors. Hazard ratios (HR) were estimated by Cox proportional regression and population attributable fractions (PAFs) estimated from the adjusted models.

There was a 5% lower risk (adjusted HR 0.952, 95% confidence interval (CI): 0.946, 0.958) of all cancers per point score of the index for men and 4% (adjusted HR 0.961, 95% CI: 0.956, 0.966) for women. The fourth versus the second category of the HLIS was associated with a 28% and 24% lower risk for men and women respectively across all cancers, 41% and 33% for alcohol-related, 49% and 46% for tobacco-related, 41% and 26% for obesity-related, and 21% for female reproductive cancers.

Findings suggest simple behavior modifications could have a sizeable impact on cancer prevention, especially for men.

## INTRODUCTION

There were over 14 million new cancer cases, and more than 30 million people living with cancer (within 5 years of diagnosis) worldwide in 2012.^1^ The overall age standardized cancer incidence rate was almost 25% higher in men than in women, with rates of 205 and 165 per 100,000, respectively.^1^ Male incidence rates varied approximately 5-fold across the different regions of the world, while those for females varied 3-fold.^1^

It has been estimated that at least a third of the most common cancers are related to lifestyle and as such are preventable.^2^ Individual modifiable lifestyle factors have been shown to be associated with cancer risk such as smoking,^3^ alcohol consumption,^3^ diet,^2^ physical activity,^2^ and anthropometry.^4^ People have a propensity to follow common behavioral patterns,^5^ and such lifestyle factors are often clustered, therefore, it seems logical to examine these lifestyle factors jointly.

There is evidence mounting on the association of patterns of behavior, or combined lifestyle factors, to cardiovascular disease^5,6^ and diabetes,^7^ and more recently, to cancer types.^8,9^ Benefits of adhering to healthy lifestyles have been quantified specifically in relation to cancer risk in a prior study which assessed the association between concordance with World Cancer Research Fund (WCRF)/American Institute for Cancer Research (AICR) overall cancer prevention guidelines and subsequent cancer risk in the European Prospective Investigation into Cancer and Nutrition (EPIC) cohort and reported a protective effect of adhering to the guidelines; nevertheless the effect varied among cancer types, showing the score worked well in some cancers but not for all cancers.^10^ Further investigations within the EPIC cohort include various health index associations with specific cancer sites (e.g., breast,^11^ colorectal,^12^ gastric^13^). In order to examine specific risk-related cancer subgroupings (i.e., alcohol-related cancers, tobacco-related cancers, obesity-related cancers, and, among women, reproductive-related cancers) within EPIC, an a priori healthy lifestyle index was created based on posited dietary components as previously proposed.^5,7,11,14^ Fiber, carbohydrates, fruits and vegetables, red and processed meats, and different fatty acids have all been posited to affect cancer risks.^2,15,16^ The healthy lifestyle index and its 5 components: smoking status, physical activity, alcohol consumption, diet, and body mass index (BMI), were used to assess associations with all cancer, and the alcohol-, tobacco-, obesity-, and reproductive-related cancer groupings.

## METHODS

### Study Population

EPIC is a prospective cohort study conducted in 23 centers across 10 European countries (Denmark, France, Germany, Greece, Italy, the Netherlands, Norway, Spain, Sweden, and the United Kingdom).^17^ The cohort of 521,330 healthy men and women were recruited from 1992 to 2000, to investigate the relationship between nutrition, dietary habits and lifestyle, and cancer incidence. Participants were aged between 25 and 70 years and enrolled from the general population, with exceptions for France (national health insurance scheme members), Utrecht and Florence (breast cancer screening participants), Oxford (health conscious, mainly vegetarian, volunteers), and some centers from Italy and Spain (blood donor participants). The rationale, study design, and methods for EPIC have been described in detail elsewhere.^17^ Ethical approval was obtained from participating centers and IARC ethics committees. All study participants gave informed consent.

### Data Collection and Follow-Up

Participants completed validated country-specific questionnaires at baseline, including interviewer-administered diet histories or self-administered semi-quantitative food frequency questionnaires to measure usual intakes.^18^ The harmonized EPIC nutrient database was used to estimate energy intake.^19^ Sociodemographic data, smoking history, alcohol consumption, and physical activity were obtained from lifestyle questionnaires, and anthropometric measurements taken, except for Oxford and France where measurements were self-reported.^17^

In Denmark, Italy, the Netherlands, Norway, Spain, Sweden, and the UK follow-up was performed through cancer registries. In France, Germany, and Greece, follow-up was performed through health insurance records, cancer/pathology registrations, and via participants and their next-of-kin. Follow-up commenced at date of enrolment and finished at date of cancer diagnosis, death, or at last complete follow-up (December 2004 to June 2010, depending on each center), whichever came first. Cancer incident cases were defined as first primary invasive tumors (coded using the 10th Revision of International Statistical Classification of Diseases).

### Cancer Subgroupings

#### Alcohol-Related Cancers

Colorectal cancer [C18–C20], female breast cancer [C50], upper aero-digestive (UADT) cancers (including cancer of the mouth [C01–C10 without C08 = salivary gland], larynx [C32], pharynx [C11–C14], esophagus [C15]), and liver cancer [C22–C24].^2,3^

#### Tobacco-Related Cancers

Upper aero-digestive cancers (including cancer of the mouth [C01–C10 without C08 = salivary gland], larynx [C32], pharynx [C11–C14], esophagus [C15]), liver [C22–C24], pancreas [C25], bladder [C67], kidney [C64, C65], cervix [C53], stomach [C16], trachea [C33], lung [C34], acute myeloid leukemia [C92], and colorectum [C18–C20].^3^

#### Obesity-Related Cancers

Esophagus [C15], pancreas [C25], colorectum [C18-C20], breast (after menopause) [C50], endometrium (lining of the uterus) [C54], kidney [C64, C65], thyroid [C73], and gallbladder [C23].^2^

#### Female Breast and Reproductive-Related Cancers

Breast (after menopause) [C50], vulva [C51], vagina [C52], cervix [C53], uterine [C54–C55], ovary [C56] and other female genital organs [C57–C58].

The original EPIC cohort comprised 521,330 men and women; 477,312 after the exclusion of participants with prevalent cancers (23,785) or missing follow-up information (4380), missing dietary or lifestyle questionnaires (6253), and those in the top or bottom 1% of the ratio of energy intake to energy requirement (9600). The present study was based on data from 391,608 men and women, following exclusions of those that were not primary malignant cancers (10,392), and those with missing data for the components of the lifestyle index (75,312), including all participants from Umea in Sweden, and Norway, where information on physical activity was not collected.

### Index Construction

#### Score for Diet

Intakes of 6 dietary factors were combined for the diet score: cereal fiber, red and processed meat, the ratio of polyunsaturated to saturated fat, margarine (as a marker for industrially produced trans-fats), glycemic load, and fruits and vegetables. The linear regression residuals of each dietary component on total energy intake were grouped into country-specific deciles and scored from 0 to 9 (inverse for red/processed meat, trans-fat, and glycemic load), with 0 being least healthy consumption (for margarine there was a non-consumers category). The individual scores were summed to a total diet score, and then categorized into quintiles.^11^

#### Score for Health Index

The overall healthy lifestyle index was determined by assigning scores of 0 to 4 to each individual variable category, for which a higher point value indicates a healthier behavior.^11^ The healthy lifestyle index ranged from 0 to 20. Healthiest behavior was defined as never smoking (never smoked = 4, ex-smokers quit > 10 years = 3, ex-smokers quit ≤ 10-years = 2, current smoking ≤15 cigarettes/day = 1, current smoking > 15 cigarettes/day = 0), low consumption of alcohol (<6.0 g/day = 4, 6.0–11.9 g/day = 3, 12.0–24.9 g/day = 2, 24.0–59.9 g/day = 1, 60+ g/day = 0), top quintile of physical activity based on recreational and household metabolic equivalent tasks (5th quintile = 4, 4th quintile = 3, 3rd quintile = 2, 2nd quintile = 1, 1st quintile = 0), a healthy BMI (<22 = 4, 22–23.9 = 3, 24–25.9 = 2, 26–29.9 = 1, 30+ = 0), and a healthy diet, that is, high in cereal fiber, with a high ratio of polyunsaturated to saturated fat, high intake of fruits and vegetables, and low in red/processed meat, margarine/trans-fat and glycemic load (5th quintile = 4, 4th quintile = 3, 3rd quintile = 2, 2nd quintile = 1, 1st quintile = 0).

### Statistical Analysis

All analyses are sex-specific. Descriptive statistics are provided by cross tabulations with medians and interquartile ranges for continuous variables, and percentages for categorical variables. Cox proportional hazard regression models were used to estimate associations between the healthy lifestyle index and risk factor related cancer groupings. Age was used as the primary time variable, with entry time defined as age at study entry, and exit time as age at diagnosis of first primary cancer or censoring (which ever occurred first).

Hazard ratios (HR) and 95% confidence intervals (CI) were stratified by center, to control for center-specific effects, and 1-year age bands (age at study entry). Models were adjusted by height (continuous), education (none/primary, secondary/technical, university, unknown), and total energy intake excluding alcohol (continuous). The healthy index was modeled as a continuous and categorical variable (≤5, 6–10, 11–15, ≥16), using the second lowest score group as the reference category as some strata contained low numbers of healthiest group. The test for trend was performed by assigning median values of each of the four categories of the index, which was then modeled as a continuous variable. Two-sided *P*-values are provided with statistical significance set at *P* < 0.05. All models were tested for and satisfied the proportional hazards assumption.

The population attributable fractions (PAFs) of cancer cases that might be associated with the lifestyle index score were estimated, with the assumption of a causal relationship, using the following equation: p(RR − 1)/p(RR − 1) + 1, where p is the proportion of the cohort without cancer in the lowest three categories of the index (<16 on the index score) and RR is the association between the exposure and cancer, estimated by the adjusted HR comparing risk in the lowest categories to the highest of lifestyle index score.

Sensitivity analyses were performed to assess the robustness of the findings. Models were run separately for Northern, Central, and Southern European based centers. The healthy lifestyle index was recalculated with the highest/healthiest score assigned to the second BMI category (22–23.9). Models were run separately for tobacco-related cancers among never smokers. Reverse causality was tested through the exclusion of those whose cancer diagnosis was within their first 2 years of follow-up.

Analyses were performed using Stata version 11.2 and SAS version 9.3.

## RESULTS

The overall cohort of 391,608 comprised 121,200 men and 270,408 women. Among men there were 10,950 first primary incident cancer cases recorded during a median follow-up time of 11.6 years and 1,339,718 accumulated person-years. Among women there were 17,564 first primary incident cancer cases recorded during a median follow-up time of 11.8 years and 3,006,119 accumulated person-years.

Table 1 shows medians, or percentages, for each component of the healthy lifestyle index, and for each covariate characteristic. Across categories of the index score, patterns are similar for both men and women, with healthy behavior being more frequent among the higher point scoring categories. Height and education are both increased among men in the higher point scoring groups; however, these patterns were not seen among women.

**TABLE 1:**
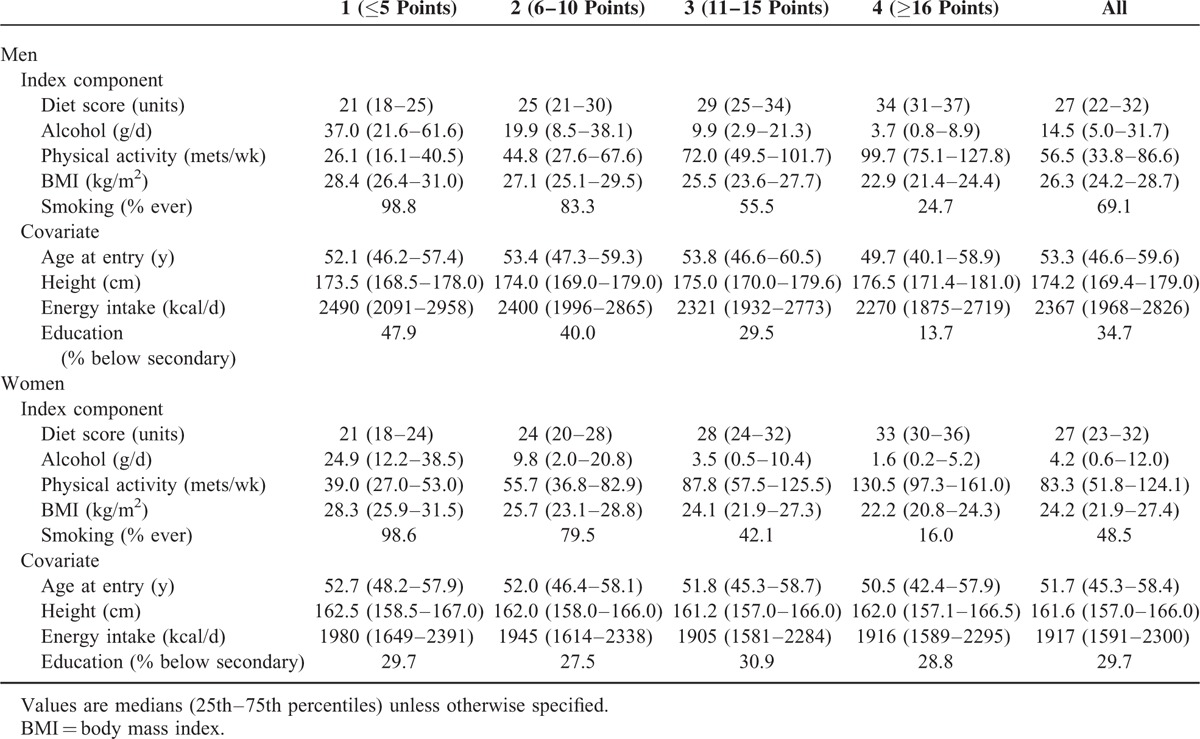
Characteristics of the European Prospective Investigation Into Cancer and Nutrition Cohort Study According to Healthy Lifestyle Index Category, 1992–2000

Table 2 shows the scoring system used for the individual components of the healthy lifestyle index, and the corresponding hazard ratios (HR) and 95% confidence intervals (CI) for each component score and cancer, adjusted for the other covariates, separately for men and women. Healthy behaviors were inversely associated to cancer risk; although not all components and score levels reached statistical significance. Specifically, among women physical activity and BMI were not associated with tobacco-related cancers. Among men, physical activity was not associated with all cancers combined, or any of the risk factor related cancer groups.

**TABLE 2:**
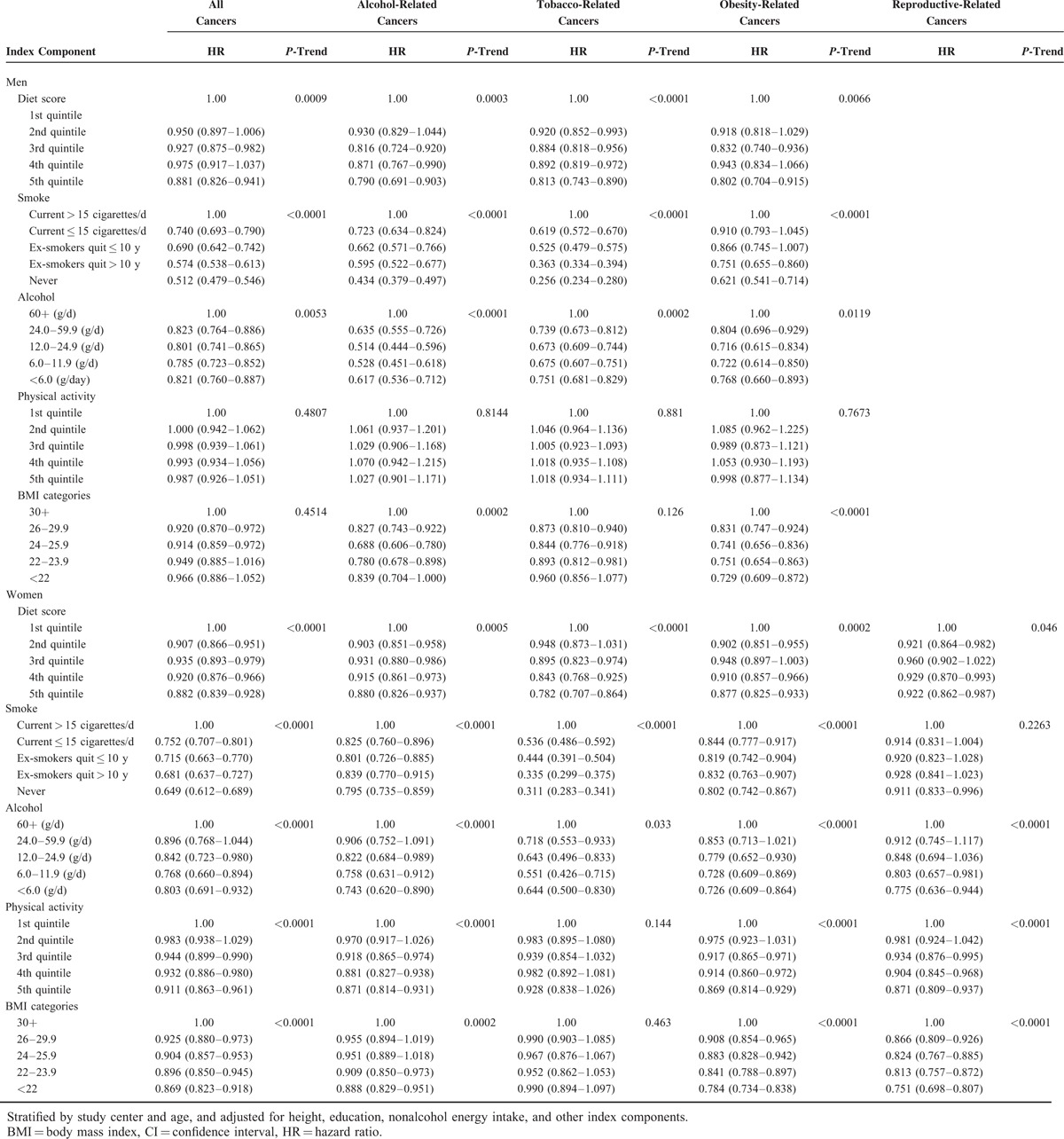
Associations for Healthy Lifestyle Index Components and Cancer Risks in the European Prospective Investigation Into Cancer and Nutrition Cohort Study, 1992–2000

Modeled as a continuous variable, there was a 5% lower risk (adjusted HR 0.95, 95% CI: 0.95, 0.96) of all cancers per point score of the index for men and 4% lower risk (adjusted HR 0.96, 95% CI: 0.96, 0.97) for women. Table 3 shows the adjusted HRs and 95% CIs for the associations between the healthy lifestyle index and cancer groupings for men and women. For men, there was a 28% lower risk of all cancer (HR 0.72, 95% CI: 0.65, 0.80); 41% lower risk of alcohol-related cancer (HR 0.59, 95% CI: 0.47, 0.75); 49% lower risk of tobacco-related cancer (HR 0.51, 95% CI: 0.43, 0.60); and 41% lower risk of obesity-related cancer (HR 0.59, 95% CI: 0.47, 0.73) in the fourth category of the index (most healthy) compared to the second (reference) category of the index score. For women, there was a 24% lower risk of all cancer (HR 0.76, 95% CI: 0.73, 0.8); 23% lower risk of alcohol-related cancer (HR 0.77, 95% CI: 0.72, 0.82); 46% lower risk of tobacco-related cancer (HR 0.54, 95% CI: 0.50, 0.61); 26% lower risk of obesity-related cancer (HR 0.74, 95% CI: 0.70, 0.80); and 21% lower risk of breast and reproductive cancers (HR 0.79, 95% CI: 0.73, 0.85) in the fourth category of the index (most healthy) compared to the second (reference) category of the index score.

**TABLE 3:**
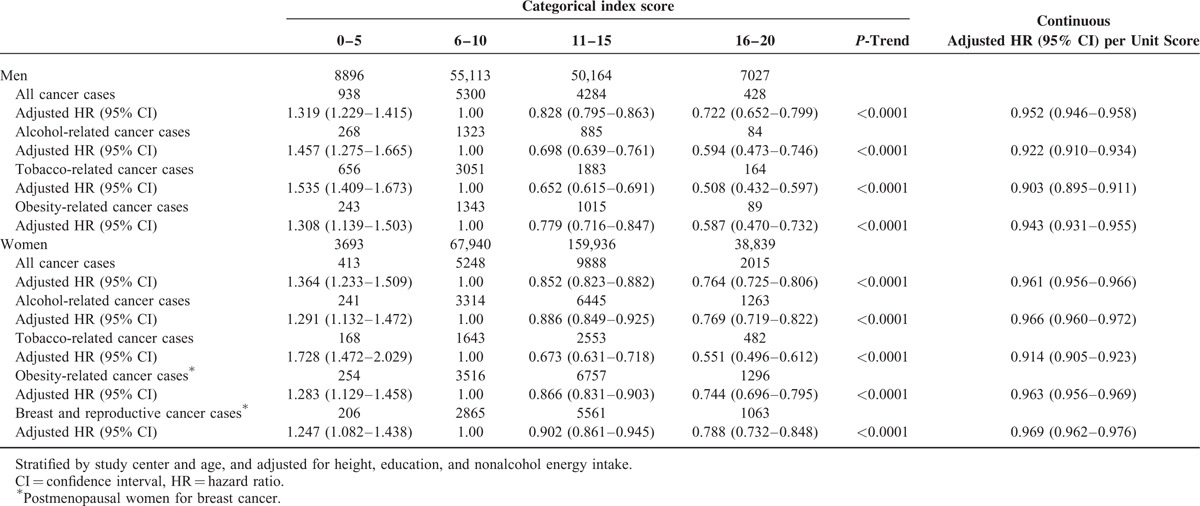
Associations for the Healthy Lifestyle Index and Cancer Risks in the European Prospective Investigation into Cancer and Nutrition Cohort Study, 1992–2000

**FIGURE 1:**
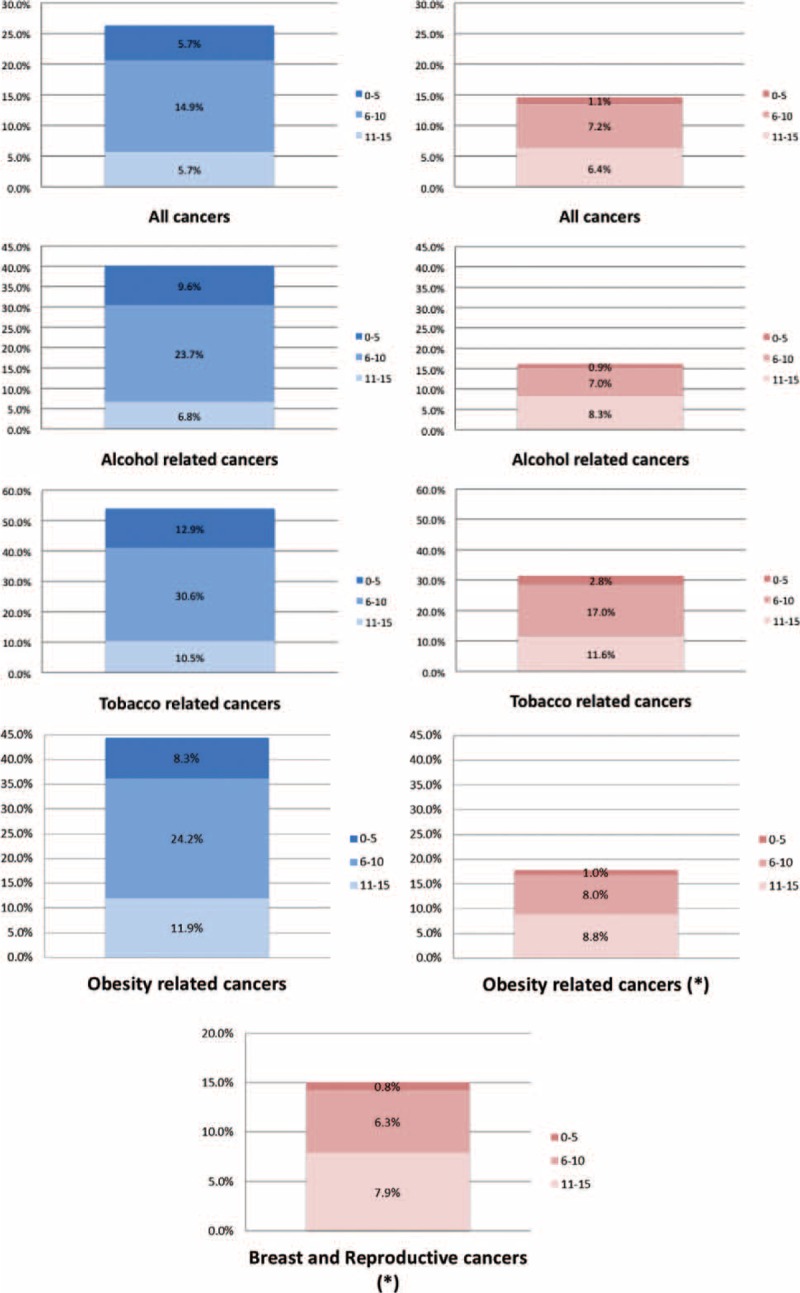
Cumulative population attributable fractions (PAFs) for healthy lifestyle index categories and cancer risks in the European Prospective Investigation into Cancer and Nutrition cohort study, 1992–2000.

PAFs were calculated for men and women to estimate the proportions of cancers that hypothetically would not have occurred if everyone had a healthy lifestyle score within the highest category of the study cohort. Figure 1 shows cumulative PAFs for all cancers combined, and for the different risk-related cancer groupings. For men, 26% of overall cancer cases could be attributed to having a lower healthy lifestyle score (below 16 on the index); while for women the estimate was much lower, with 15% of cases that could be attributed to a lower healthy lifestyle score. Among both men and women, the greatest PAFs were seen for tobacco-related cancers (54% and 33%, respectively).

Sensitivity analyses were performed to assess the robustness of the findings. Models were run separately for Northern, Central, and Southern European based centers and no material difference in results (not shown) was observed. Reverse causality was tested through the exclusion of those whose cancer diagnosis was within their first 2 years of follow-up; these results (not shown) did not differ from those of the entire cohort. For tobacco-related cancers, the healthy lifestyle index still showed a protective effect among never smokers: there was a 2% lower risk (adjusted HR 0.98, 95% CI: 0.95, 1.01) per point score of the index for men and 3% (adjusted HR 0.97, 95% CI: 0.95, 0.99) for women. None of the sensitivity analyses materially altered results or changed interpretation of findings.

## DISCUSSION

This study based on a large prospective cohort has found a lower risk of cancer in men and women with healthier lifestyles. The findings suggest modification of behavior resulting in a single point increase in the healthy lifestyle index score (HLIS) corresponds to a 5% and 4% lower overall cancer risk, for men and women, respectively, with even lower risks associated with alcohol- and tobacco-related cancers. If the associations were causal, 26% of the cancer cases in men, and 15% in women, would have been prevented if the entire cohort had been in the highest scoring category of the healthy lifestyle index.

All individual components of the healthy lifestyle index were associated with cancer risk for women; for men, all components except physical activity and BMI, which did not reach conventional significance. However, the physical activity component used in this study combines a measure of recreational activity with household activity, which is notably lower among men than women within the EPIC cohort.

Findings for the present study are consistent with previous reported studies, which found protective associations between cancer and healthy lifestyle indexes. One study based on the EPIC cohort found a lower cancer risk for men and women in the highest scoring category of a score based on adherence to WCRF/AICR recommendations^2^ compared to the lowest category (HR 0.84, 95% CI: 0.72–0.99; and HR 0.81, 95% CI: 0.72–0.91, respectively).^10^ The components of this index for all cancer were: degree of adiposity, physical activity, foods that promote weight gain, plant foods, red and processed meat, alcohol intake, and breastfeeding.^10^ Similarly, a Women's Health Initiative study used the American Cancer Society Nutrition and Physical Activity Cancer Prevention Guidelines to assess cancer risk in postmenopausal women and found lowest to highest category of their score had lower risk of any cancer (HR 0.83, 95% CI: 0.75–0.92).^20^ Whereas, a Framingham Offspring cohort study used a score based on seven components: body fatness, physical activity, foods that promote weight gain, plant foods, animal foods, alcohol, and food preservation/processing/preparation, to assess adherence to WCRF/AICR recommendations^21^; this study found no association with the overall score and obesity-related cancer risk. A simpler index was used in the women only E3N cohort in France, which used the five lifestyle factors: BMI, physical activity, smoking, alcohol consumption, and fruit and vegetable consumption, and found lower risk of all-site cancer for women associated with higher healthy lifestyle index categories (highest compared to lowest HR 0.81, 95% CI: 0.73–0.89).^22^ A postmenopausal breast cancer specific healthy index previously used for the EPIC cohort included identical components to the present study, although the factors included within the diet component differed slightly; this combined seven dietary factors: cereal fiber, folate, the ratio of polyunsaturated to saturated fat, fatty fish (as a marker for omega-3 fatty acids), margarine (as a marker for industrially produced trans-fats), glycemic load, and fruits and vegetables.^11^ This study found breast cancer risk was inversely associated with a high index score (fourth vs second categories HR = 0.74; 95% CI: 0.66–0.83).

The present study has several strengths, including the large size and prospective design of the cohort, and the long follow-up for EPIC participants. Detailed dietary and lifestyle information was collected before cancer diagnosis, thereby eliminating risk of recall bias, and ensuring any misclassification for these variables would most likely be nondifferential, underestimating the observed associations. Nevertheless, as information was not available for trans-fat in the dataset, margarine was used as an indicator; this food group has been described in the literature as the main source for industrially produced trans fatty acids.^23^ Margarine has been related to plasma elaidic acid (a biomarker for trans-fat intake) within EPIC showing the strongest correlation among numerous food groups investigated.^24^

Even though the index components were equally weighted, there is likely to be unintentional weighting because some of the factors are “recommended” items or items perceived as positive such as physical activity; while others are “moderation” items, viewed as negative such as smoking. Index components which are recommended are those behaviors which are encouraged, and as such, they may be weighted unintentionally through their promotion more so than discouraged behaviors, like the negative moderation components.^25^

EPIC participants are volunteers, and as such this is not an ordinary population-based cohort. Participants are more likely to be healthier than the general population, and therefore the estimates may be attenuated, providing an underestimation of PAFs that could be expected in the general population. A further consideration in the interpretation of results is that all PAF estimates are based on an assumption that the relationship between exposure and cancer is actually a causal relationship.

In order to avoid high correlations within and between food groups, the number of indicator foods for an index should be restricted to as few as possible, while still capturing risk-related components. Many healthy lifestyle indices have been used in recent studies to assess cancer risk, and many of these studies have had similar results, further validating the use of scores and their underlying concept of combined lifestyle modifications. Nevertheless, prospective work should investigate and compare these indices for a balance of simplicity and effectiveness to guide the future research in this area.

In conclusion, we evaluated the association between a healthy lifestyle index (including healthy diet, avoidance of smoking and alcohol consumption, moderate and vigorous-intensity physical activity, and low BMI) and the risk of cancer among men and women, and found a protective association of a healthy lifestyle among all cancer groupings. The combined healthy lifestyle index was overall more strongly associated with cancer risk for men. Cancer is a complex multifactorial disease; nevertheless, these findings add further weight to the suggestion that simple behavior modifications could have a major impact on cancer incidence. Cancer prevention policies should include strategies to engage men and women in lasting healthy diet and lifestyle habits.
